# Electrochemical Behaviour of Ti/Al_2_O_3_/Ni Nanocomposite Material in Artificial Physiological Solution: Prospects for Biomedical Application

**DOI:** 10.3390/nano10010173

**Published:** 2020-01-19

**Authors:** Alla Vorobjova, Daria Tishkevich, Dmitriy Shimanovich, Maxim Zdorovets, Artem Kozlovskiy, Tatiana Zubar, Denis Vinnik, Mengge Dong, Sergey Trukhanov, Alex Trukhanov, Valery Fedosyuk

**Affiliations:** 1 Department of Micro- and Nanoelectronics, Belarusian State University of Informatics and Radioelectronics, 220013 Minsk, Belarus; vorobjova@bsuir.by (A.V.); shdl@tut.by (D.S.); 2 Laboratory of Magnetic Films Physics, Scientific-Practical Materials Research Centre of National Academy of Sciences of Belarus, 220072 Minsk, Belarus; fix.tatyana@gmail.com (T.Z.); sv_truhanov@mail.ru (S.T.); truhanov86@mail.ru (A.T.); fedosyuk@physics.by (V.F.); 3 Laboratory of Single Crystal Growth, South Ural State University, 454080 Chelyabinsk, Russia; denisvinnik@gmail.com; 4 The Institute of Nuclear Physics, Almaty 050032, Kazakhstan; mzdorovets@gmail.com (M.Z.); artem88sddt@mail.ru (A.K.); 5 L.N. Gumilyov Eurasian National University, Nur-Sultan 010008, Kazakhstan; 6 Ural Federal University named after the First President of Russia B.N. Yeltsin, 620075 Yekaterinburg, Russia; 7 Department of Resource and Environment, Northeastern University, Shenyang 110819, China; mg_dong@163.com

**Keywords:** nickel-alumina, nanocomposite, electrochemical deposition, potentiodynamic polarization, cyclic voltammetry, corrosion resistance, biomedicine

## Abstract

Inorganic-based nanoelements such as nanoparticles (nanodots), nanopillars and nanowires, which have at least one dimension of 100 nm or less, have been extensively developed for biomedical applications. Furthermore, their properties can be varied by controlling such parameters as element shape, size, surface functionalization, and mutual interactions. In this study, Ni-alumina nanocomposite material was synthesized by the dc-Ni electrodeposition into a porous anodic alumina template (PAAT). The structural, morphological, and corrosion properties were studied using x-ray diffraction (XRD), scanning electron microscopy (SEM), atomic force microscopy (AFM), and electrochemical techniques (linear sweep voltammetry). Template technology was used to obtain Ni nanopillars (NiNPs) in the PAAT nanocomposite. Low corrosion current densities (order of 0.5 µA/cm^2^) were indicators of this nanocomposite adequate corrosion resistance in artificial physiological solution (0.9% NaCl). A porous anodic alumina template is barely exposed to corrosion and performs protective functions in the composite. The results may be useful for the development of new nanocomposite materials technologies for a variety of biomedical applications including catalysis and nanoelectrodes for sensing and fuel cells. They are also applicable for various therapeutic purposes including targeting, diagnosis, magnetic hyperthermia, and drug delivery. Therefore, it is an ambitious task to research the corrosion resistance of these magnetic nanostructures in simulated body fluid.

## 1. Introduction

Currently, the use of nanomaterials in biomedicine reached significant achievements in the diagnostics, prevention, and therapy of a large number of illnesses. This promising area uses various types of nanostructures (nanowires, nanotubes, nanopillars, nanoparticles, etc. [1,2,3,4,5,6]). Magnetic nanostructures find various applications in biomedicine, such as targeted drug delivery controlled by an external magnetic field [7,8,9]. Targeted drug delivery is a developing and promising technique for the treatment of cancer. This technique is based on the attachment of a therapeutic drug agent to the functional groups of the magnetic material nanostructure, its injection into the patient’s body (blood), and further transportation to the problem region. After the exposure to the tumor, the magnetic nanostructures are deleted from the blood system by an external magnetic field [4]. Nanocomposite materials are widely used in the field of different biomedical applications such as developing of materials to replace/regenerate tissues starting from tissue engineering with mesenchymal stem cells (MSCs) and biomimetic scaffolds [10], bone therapeutic applications [11,12,13], especially in regards to pathological fractures/injuries such as the one derived from osteoporosis [14]. The role of inflammatory conditions in rehabilitation with biomaterials should be noted, as inflammatory phenomena may trigger the oxidative stress that would take advantage of perioperatory therapy [15].

Template synthesis is one of the most convenient and promising methods for nanostructures formation. Templates based on porous anodic alumina are commonly used for the fabrication of new nanostructured composites based on nanoscale structures. Such nanostructures are widespread due to their unique chemical and physical properties [16,17,18,19,20,21,22]. The full extent of anodic alumina template usage in forming nanostructured materials is discussed in the review article [17].

At this stage of nanotechnologies progress, the mechanisms of using arrays of nanoelements (nanodots, nanowires, nanopillars) as sensitive elements of biological sensors are actively underway. Various types of Faradaic and non-Faradaic electrochemical impedance biosensors are offered, differing in methods of design miniaturization [23,24]. The defined component reacts with the sensitive layer directly on an electrode surface (Ti/PAAT/Ni, Ti/PAAT/Ni/NiO electrodes in our case) or in the volume of the solution layer close to the electrode. In such biosensors, the electrodes may have regular micro-sensor dimensions. Based on this, the most available (at the moment) nanoelement biosensor manufacturing option is the integration of nanoelements arrays with planar electrodes of biosensors. State-of-the-art electrochemical biosensor platforms consist of functionalized metal electrodes. By replacing each metal electrode with a nanoelements array of the same area, it is expected to increase the sensitivity, and the detection probability [25]. In addition, the formation of the upper electrode of the sensor in the shape of a nanoporous structure with the specified pore size allows achieving the required level of selectivity and eliminating adsorption of interfering components on the sensitive layer [26]. For instance, alumina coated capacitive detectors were developed for the detection of DNA, constructed with typical CMOS materials. Real-time detection of pathogenic microorganisms is a developing area of research, particularly regarding microorganisms that present a major threat to human health [27].

It is known that the electrochemically deposited Ni films were generally used to enhance the mechanical and electrochemical characteristics of different alloys. However, many articles concern the corrosion behavior of conventional bulk coatings and alloys based on Ni [28,29,30,31]. The passive behavior of such Ni in acidic and alkaline solutions was discussed in several papers [32,33]. The processes of passivation of Ni, both in acidic and alkaline solutions, are due to the creation of a variable composition layer of oxides on its surface. The corrosion properties of Ni have been substantially investigated in aqueous solutions (acid and alkaline), but only some papers have been devoted to the features of their corrosion behavior in a physiological medium.

The studies on the corrosion properties of Ni-based nanocomposites are also limited [34,35]. The electrochemical behavior of Ni nanopillars (NiNPs) in porous anodic alumina template (PAAT) or Ni nanopillars alone in artificial physiological solution (APS) (such as 0.9% NaCl aqueous solutions) has not been examined. The corrosion protective coatings from nanocrystalline Ni thin films are described only in a few articles [36,37,38].

One of the recently discovered options surrounding nanostructured composite materials application is connected with their inherent properties of adsorption and reaction control in the adsorbed layers. The set of these properties defines their surface functionality. The surface functionality is a basis for the use of nanocomposite materials in many areas, in particular, its biomedical application [39,40,41,42,43,44,45]. Unfortunately, the data on the reactions observed during the contacts of nanomaterials with organic and inorganic matter is extremely limited and contradictory. Therefore, the major problem of nanotechnologies is the problem of ensuring adequate control of functional properties of nanostructures surface, including in accordance with external conditions.

At present, scientific research in the field of composite nanomaterials, biomedicine, filtration membranes, catalysts, sensors, drug delivery, etc., assumes not only their technological performance but also the assessment of potential capabilities of these materials in the actual-use environment. From this point of view, studying the functional properties of composite materials surface depending on the medium (external conditions) is a very prospective task.

In view of the aforesaid, the aim of this research is to study the electrochemical behavior of Ni nanopillars nanocomposite material (NiNPs) in porous anodic alumina template (PAAT) in APS (0.9% sodium chloride solution).

## 2. Materials and Methods

Ni-alumina nanocomposite material was formed by dc- Ni electrodeposition into a porous anodic alumina template (PAAT) [46]. The composite represents the array of Ni nanopillars (NiNPs) in a dielectric template (NiNPs/PAAT).

The samples constitute of thin structures of Ti and Al on silicon/silicon oxide substrates. Al films with poly-crystalline structure and 3000 ± 50 nm thickness and Ti films with 450 ± 50 nm thickness were deposited via electron-beam technique. The 01-NE-7004 ORATORIYA-9 equipment was used for film deposition. Ti layers were sputtered at 5.0 × 10^−4^ Pa vacuum, the temperature of substrate −300 °C and the deposition speed was (2.0 ± 0.2) nm/s. Films of Al were obtained under the following conditions: vacuum degree −1.4 × 10^−4^ Pa, temperature −150 °C. A quartz sensor was used for controlling the film’s deposition rate.

Highly ordered PAATs were produced by two-step electrochemical anodization inn 4% H_2_C_2_O_4_ in a potentiostatic mode at 40 V and 14 °C electrolyte temperature. A two-electrode cell was used for the anodizing process. A steel sheet was used as a cathode. Electrolyte mixing was carried out by a magnetic stirrer.

Most studies on metals electrochemical deposition in PAAT concern sedimentation into the pores of a free membrane (with through pores), thicker than 20 µm. The deposition into the PAAT on a dielectric or semiconductor substrate remains a problematic technological operation because of the presence of a barrier layer at the porous oxide bottom.

Therefore, after anodizing is carried out, the process of removal (or thinning) of a barrier layer at the bottom of PAAT pores is essential. Usually, the methods including several technological operations are used: in the final stage of the anodizing process, the voltage (or current density) is reduced gradually with a low rate (or stepwise). Then chemical etching of the residual barrier layer in aqueous solutions of acid is carried out [47,48,49].

**Figure 1:**
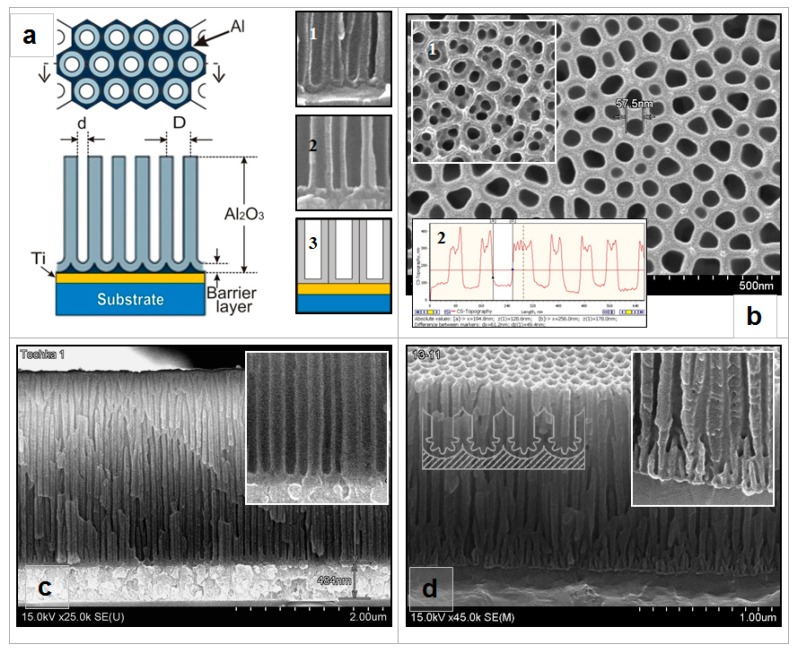
(**a**)—Scheme of porous anodic alumina template (PAAT). The insets show the scanning electron microscopy (SEM) picture of a cross-section of the sample before (1) and after the etching of barrier layer (2), the scheme of this fragment cross-section (3). (**b**)—Surface view and atomic force microscopy (AFM) profile (inset 2) of the experimental sample after barrier layer etching. Inset 1 shows the SEM image of this sample before the etching. (**c**,**d**)—cross-section views of the experimental sample before Ni deposition into PAAT pores.

In this work, we adopted a different approach. After anodizing of the Al film and obtainment of the Ti film, the anodizing voltage was not reduced but remained invariant within 20–25 min. During this period a partial electrochemical barrier layer dissolution at the oxide pores bottom solely, i.e., thinning, takes place, and anodizing of Al residues between oxide cells happens. In the course of thinning not only was the thickness of the barrier layer reduced, but also the morphology of the surface of the barrier layer changed. In this manner, Ti is not anodized and not dissolved through the porous oxide with the remaining barrier layer, and the internal Ti film-to-oxide interface becomes planar. Figure 1 presents a scheme of the PAAT (a) and scanning electron microscopy (SEM) images of the surface of the sample (b) and cross-section (c,d) before Ni deposition into PAAT pores.

Chip SEM photos of PAAT samples after etching of a barrier layer at the oxide bottom by the offered method and by an anodizing voltage reduction method are given in Figure 1c,d, correspondingly. On insets (the enlarged images of a sample fragment next to the internal Ti film-to-oxide interface) it is clearly visible how the interface changes: in the first case it becomes planar, and in the second the regularity of the pores near this border is broken. The thickness of this zone can be about 200 nm. The other part of PAAT remains quite regular, but as the deposition of metal into the PAAT pores by a direct current begins at the bottom of the pores, the access of metal ions to this border may be complicated.

Just before the Ni electrodeposition, the selective chemical etching of the residual barrier layer was performed in a 4% aqueous solution of ortho-phosphoric acid (H_3_PO_4_) at a temperature of 30 °C for 30–40 min. When selective chemical etching is carried out, the barrier layer thickness decreases simultaneously at the bottom and walls of the pore approximately at an identical rate. In sum, a PAAT with pass-through pores on the Ti film bottom is formed. This template has the following characteristics: PAAT height −(1.3–2.5) ± 0.05 µm, pore diameter −(50–75) ± 10 nm (depending on the duration of the barrier layer etching), inter-pore distance 105 ± 10 nm. The PAAT with pores on the underlying Ti film is an electrode for electrodeposition and performing tests. The process of the removal of the barrier layer at the bottom of the PAAT pores (thinning) was reported, in detail, in [5,50].

The electrodeposition of Ni into PAAT was performed in a two-electrode cell with an auxiliary electrode (graphite plate) at a constant potential (direct current), dc-deposition by the potential sweeping at a 20 mV/s speed from 0 to a potential from −(1.8 to 2.2) V and then, at this potential value for (3.0–10) min.

Galvanostat/Potentiostat power supply P-5827 M was used for anodization, etching of barrier layer and metal electrodeposition operations controlling. When carrying out electrochemical processes in a two-electrode cell it is possible to use ordinary power supplies, which would be the option most suited to industrial conditions.

Ni was electrodeposited from the following electrolyte (in g/L): (140) NiSO_4_ × 7H_2_O + (30) NiCl_2_ × 6H_2_O + (25) H_3_BO_3_ + (60) Na_2_SO_4_. NiSO_4_ and NiCl_2_ are sources of nickel ions, H_3_BO_3_ is the stabilizer of solution acidity, NiSO_4_ is introduced to limit electrolyte corrosion activity. The electrolyte pH was determined and adjusted at the 5.2 level by Hanna HI83141 pH-meter by adding NaOH. The mixing of the electrolyte was performed with a magnetic stirrer. The temperature of the electrolyte was 20 °C.

The microstructure (topological) characteristics of samples were examined using Hitachi S-4800 SEM and Nanotop-206 atomic force microscopy (AFM). Prior to SEM and AFM studies, the samples were etched in a solution for alumina selective etching. The operation of fractional (or complete) PAAT etching was performed for a more precise estimation of the diameter of nanopillars and the PAAT filling degree. Further, the alumina boundaries of the pores and cells are more distinctly visible. The entire oxide etching is needed also to determine the structural elements’ height and diameter. Selective chemical dissolution was used for alumina removing: CrO_3_ (20 g/L) + 85% H_3_PO_4_ (35 mL/L), the temperature—(80 ± 2) °C, etching duration—3 min.

AFM and SEM images analysis were performed by the software “SurfaceXplorer” document suite. Moreover, surface SEM images were processed using the Image J editor and the OriginalPro8 program, which allow determining the individual element size in the image. The distribution histograms of the elements’ diameter were also obtained.

The X-ray diffraction (XRD) technique was used to investigate the phase composition of samples. Diffractometer DRON2 with Cu Kα radiation (λ = 0.154056 nm) was used.

The composite material electrochemical behavior investigations were carried out by potentiostat-galvanostat (AUTOLAB PGSTAT302n). NOVA software (version 1.10) was utilized for data processing. A characteristic three-electrode cell with a reference electrode Ag/AgCl was used. The auxiliary electrode was a platinum rod.

The electrochemical actions that occur on the Ti/PAAT/Ni composite material surface and the interfaces on the Si/SiO_2_ substrate (a working electrode) were studied by potentiodynamic polarization technique and cyclic voltammetry. The linear polarization method or linear sweep voltammetry (LSV), a NOVA software term, was applied for measuring the corrosion currents and potentials in a potentiodynamic mode (linear or cyclic potential-current sweep curves). The samples were kept without polarization in the solution for 2–3 min before the registration of polarization curves. In this instance, the dependence of open circuit potential (OCP) on time was recorded. The starting value of the potential was automatically adjusted at 100 mV lower relative to OCP. Linear sweep voltammograms were registered in the range of potentials −450 to +500 mV(Ag/AgCl) and potential scanning rates over the range of 1–100 mV/s. Cyclic voltammograms were recorded in the potential range from −1500 to +2000 mV(Ag/AgCl) and potential scanning rates over the range of 1–100 mV/s (0.01–1.0 V/s). The studies of corrosion resistance were carried out in physiological solution (PS) (0.9% NaCl). The PS (PS—NaCl—8.5 g, the water (distilled)—1000 mL) is analogical to the «Tyrode» solution which is a simulated body fluid. In bio- and medical practice, various biological tests are based on PS for microbiological studies (assays) [51]. Besides, the PS is used in the electrochemical behavior analysis of different biological sensors materials in a synthetic biological solution including «Tyrode» solutions [52].

The electrolyte acidity was 6.14 ± 0.01 pH. The measurements were performed at room temperature (20 ± 2 °C) without mixing. All of the studied samples’ surface areas were identical and equal to 0.6 × 0.6 cm^2^.

Initially, all layers were investigated separately: Si/SiO_2_-Ti, Si/SiO_2_-Ti/PAAT (before and after barrier layer etching). Thus, some groups of samples were produced for the electrochemical studies: Si/SiO_2_ (substrate)-Ti/Si/SiO_2_ (substrate)-Ti/PAAT and Si/SiO_2_-Ti/PAAT/Ni (further in the text Ti/PAAT/Ni electrode, without Si/SiO_2_). Such sample composition is most convenient for various studies (electrical, magnetic, thermodynamic, and electrochemical), as Ni nanoparticles (in our case nanopillars) are rigidly fixed in an inert matrix. In addition, they are arranged on a conductive electrode and on an electronics standard substrate. When specific use of NiNPs alone is required, for example, in drug delivery systems, they are easily separated from the alumina template and from the substrate by selective chemical etching.

## 3. Results and Discussion

### 3.1. Microstructure (Topological) Characteristics of Ni–PAAT Composite

**Figure 2:**
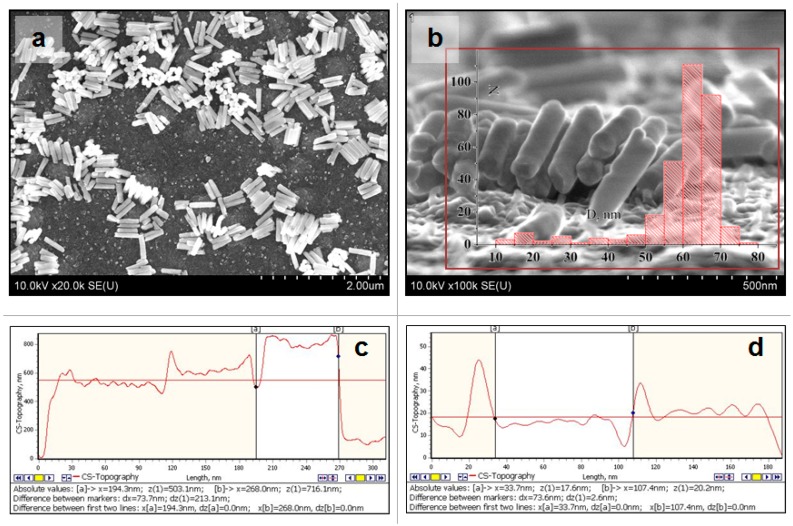
(**a**,**b**)—SEM images and histogram of the sample, obtained in dc-deposition of Ni at a constant potential −1.8 V within 5 min, after alumina selective chemical etching, (**c**,**d**)—AFM profiles of this sample surface for estimating the NPs diameter.

Figure 2 depicts the surface SEM pictures (a,b) and AFM profiles (c,d) of experimental samples after Ni electrochemical deposition into PAAT at dc-deposition mode, at constant potential −1.8 V within 5 min. Inset in Figure 2b shows the histogram of NiNPs diameter distribution.

From these photos and profiles, it is visible that at dc-deposition of Ni into the PAAT pores dense equidimensional nanopillars of Ni (NiNPs) with a smooth surface are formed. The height of NiNPs depends on the deposition time taken for NiNPs to be formed according to the ordinary mechanism of electrochemical sedimentation—bottom-up (from a pore bottom). The free growth of the high-density NiNPs array at dc-potential mode shows that the layer of Ti is conductive and the PAAT pores are opened to the film of Ti.

NiNPs topological characteristics medium values received from SEM and AFM pictures analysis are presented in Table 1.

The pore distance (and the length between the NiNPs) was estimated by the equation D_cell_ = *k* × U_anod_ = 2.6 nm/V × 40 V = 104 nm [47]. The cell diameter was about 100 nm. This characteristic was the same (100 nm) as all samples were obtained with the same anodizing voltage (U_anod_).

Thus, this process for the obtaining of composite material, consisting of two-step anodization and PAAT etching, allows producing a thin-film template with a highly ordered microstructure. This makes it possible to improve the regimes of metal electrodeposition. The degree of pore filling by metal is increasing, above all near the barrier layer (at the interface with Ti), where pores are nearly 100% filled.

In case a two-electrode cell is used, the electrochemical dc-deposition of Ni is accomplished at a higher potential relative to equilibrium one (−0.8 V to −1.6 V) or more. Cathode potential enhancement results in more intensive hydrogen evolution in an aqueous solution of an electrolyte. Bubbles of hydrogen are generated on the surface of the oxide and close (blocking) the majority of the oxide pores. Therefore, metal is not deposited in all pores and the final percentage of pore filling is reduced to 75–85% (on sample surface). Figure 2 also demonstrates that, as a result of alumina selective etching, NiNPs of equal size and shape can be separated to form various samples (probes).

**Figure 3:**
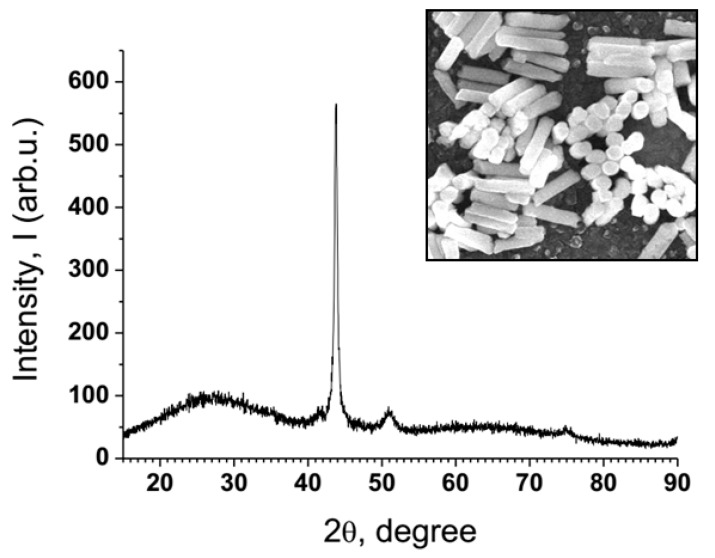
X-ray diffraction (XRD) pattern of the NiNPs nanocomposite material in PAAT.

The crystal structure of Ni-PAAT nanocomposite material was investigated by XRD technique. The XRD pattern of NiNPs–PAAT is presented in Figure 3.

The XRD spectrum with a main peak suggests a material with a crystal structure. The nickel phase has a face-centered cubic lattice (fcc) (according to powder diffraction data—01-1260J JCPDS—Joint Committee of Powder Diffraction Standards). The Al_2_O_3_ template is amorphous to X-rays (25.89°). Except for the main peak with orientation (111), there are two weaker peaks with a crystal orientation: (200) and (220). These orientations are characteristic of electrodeposited Ni into a PAAT [53].

The main direction of growth of Ni in the Al_2_O_3_ template for Ni-PAAT nanocomposite is (111) orientation. The other peaks are of lower height than the main peak. A high Ni degree of crystallinity and relative orientation along the main direction of crystallite growth are shown. The presence of other peaks of low intensity indicates a small number of crystallites with a different growth orientation.

By processing the XRD data, the average size of crystallites was calculated using the Debye–Scherrer equation:

(1) $$d\;=\;\frac{k\lambda }{B\cdot \cos\vartheta }$$

where d is the grain size, *k* is equal to 0.90–0.94 for FWHM (full width at half maximum) of spherical crystallites with cubic symmetry, *λ* is the wavelength of X-rays (*λ* = 0.154056 nm in our case), *B* is FWHM and *ϑ* is the half diffraction angle of the peak.

The Ni crystallites’ average sizes in PAAT were determined by the Debye-Scherer equation and are presented in Table 2.

The NiNPs crystallite size (~18 nm) is smaller than the mean NiNPs diameter (~70 nm). The crystallites with another size (9 nm and 20 nm) account for only 12% of the content, therefore the Ni-PAAT composite is a poly-nanocrystalline material.

### 3.2. Potentiodynamic Polarization Measurements

Initially, the interfaces of a Ti/PAAT electrode after and before barrier layer etching were investigated. The experiments were performed after corrosion potentials stabilization.

**Figure 4:**
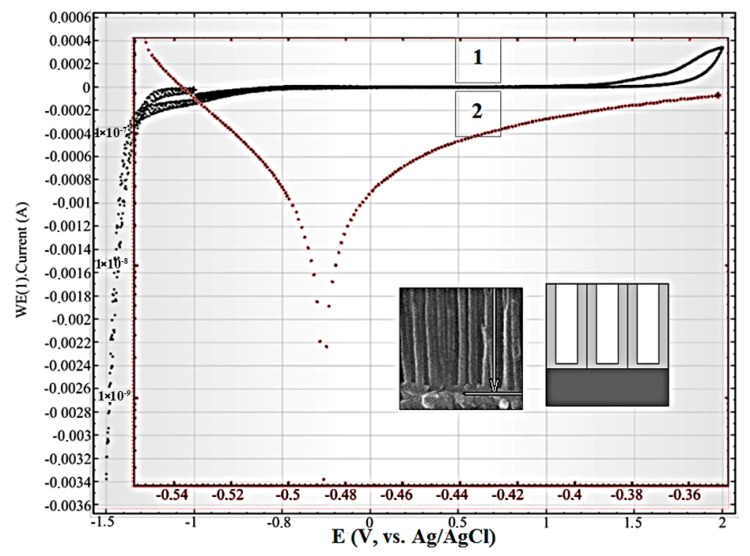
Cyclic (1) and linear sweep (2) voltammograms of the Si/SiO_2_/Ti sample in the 0.9% aqueous solution of NaCl at a potential scanning rate of 0.1 V/s.

In Figure 4, the cyclic polarization curve (1) and potentiodynamic polarization curve (linear sweep voltammograms) (2) are shown for the Ti film after the removal of the barrier layer at the bottom of the pores. On insets, the scheme and SEM cross-section view of this sample part is depicted. In this sample, the electrolyte interacts with the Ti layer through the holes at the bottom of the pore in alumina.

**Figure 5:**
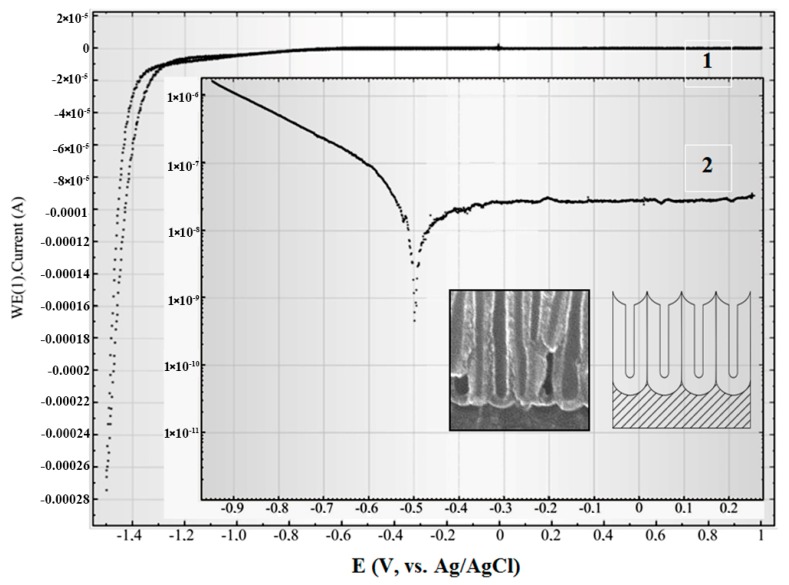
Cyclic (1) and linear sweep (2) voltammograms of Ti/PAAT sample in the 0.9% aqueous solution of NaCl at a potential scanning rate of 0.1 V/s.

Figure 5 shows the cyclic (1) and linear sweep (2) voltammograms for the Ti/PAAT sample before selective etching of the barrier layer. The insertions demonstrate the scheme and cross-section SEM view of the Ti/PAAT sample part. Actually, this sample is the PAAT—solution interface, and consequently describes the electrochemical properties of anodic alumina.

The form of cyclic polarization curves for these samples shows, that at this concentration of sodium chloride in the electrolyte, the anode currents are actually not observed. The current close to zero in the wide potentials range from −1.0 to +1.0 V (Ag/AgCl) demonstrates a lack of any redox reactions on the surfaces of Ti and PAAT electrodes.

From the linear sweep voltammograms 2 in Figure 5 the passivation parameters of the PAAT (Al_2_O_3_) were determined: start passivation potential (E_sp_ = −0.5 V (Ag/AgCl)), complete potential of passivation (E_cp_ = −0.37 V (Ag/AgCl)), passivation current (I_pass_ = 28 nA) and passivation current density (J_pass_ ~ 0.078 µA/cm^2^). So, in the given range of potentials alumina is passive, therefore it introduces the negligible contribution to whole current of corrosion.

**Figure 6:**
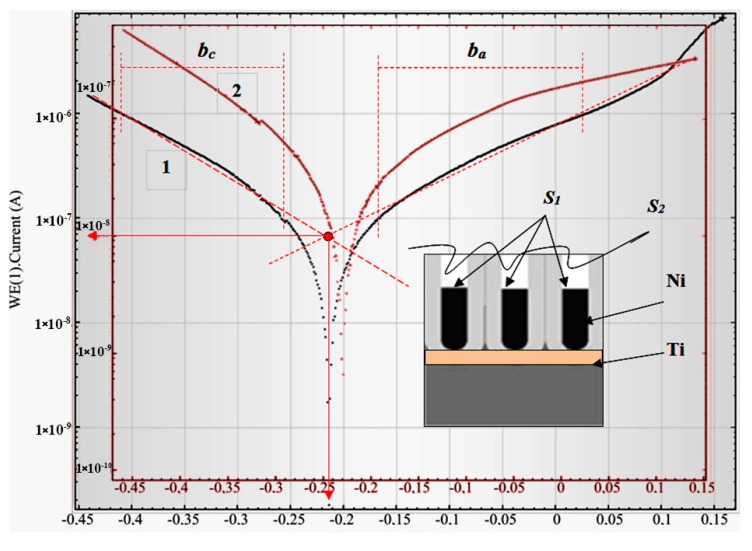
Linear sweep voltammograms of the Ti/PAAT/NiNPs sample in the 0.9% NaCl aqueous solution at a potential scanning rate of 0.1 V/s: 1—the fourth cycle of polarization, 2—the second cycle of polarization.

**Figure 7:**
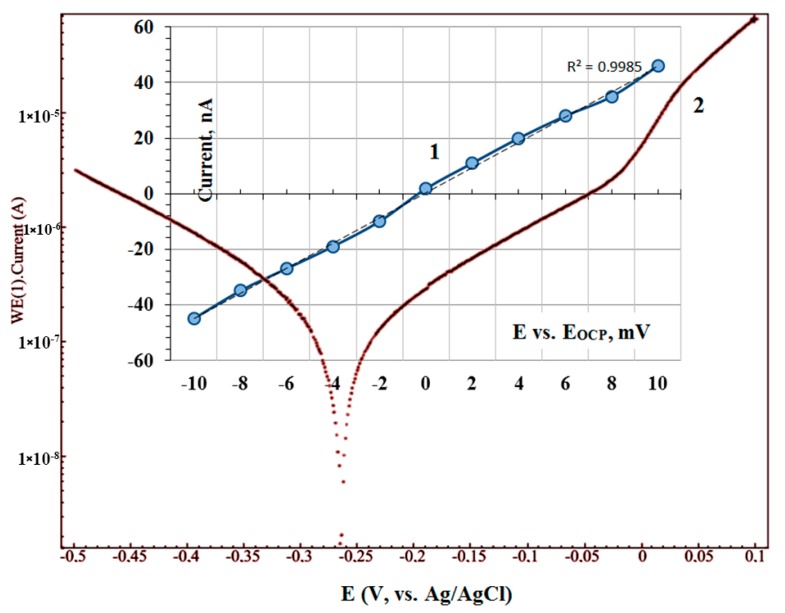
Linear sweep voltammogram of Ti/PAAT/Ni sample after stabilizing the free potential of corrosion (2). In the insert—a small part of this voltammogram within the potential range from −10 to 10 mV (curve 1).

**Figure 8:**
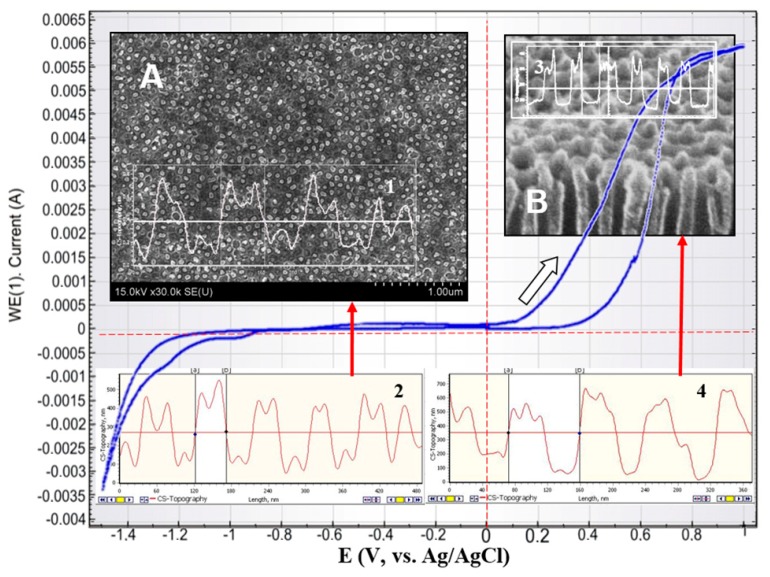
Cyclic voltammogram of the Ti/PAAT/Ni sample in the 0.9% aqueous solution of NaCl at a potential scanning rate of 0.1 V/s. In the insets: (**A**)—top view and AFM-profiles (1,2) of the experimental sample (NiNPs in PAAT) after alumina partial etching, (**B**)—cross-section views and AFM-profiles (3,4) of the experimental sample (PAAT) before NiNPs deposition into Al_2_O_3_.

**Figure 9:**
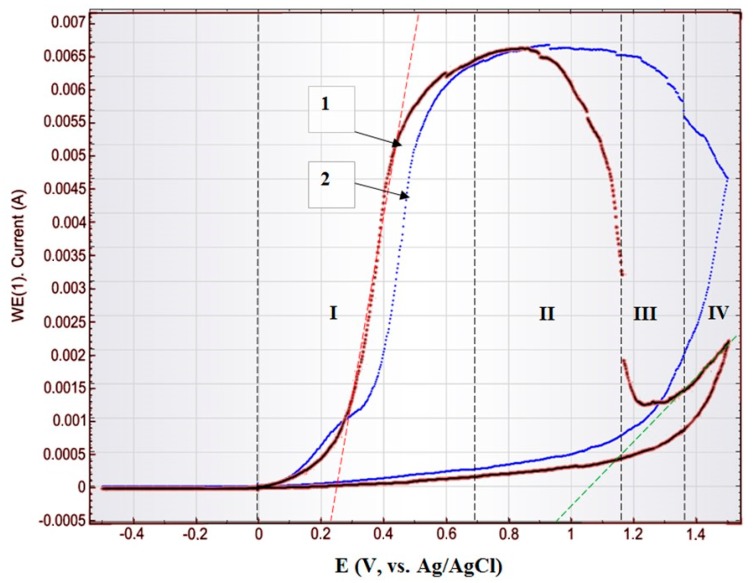
Cyclic voltammograms of the Ti/PAAT/Ni electrode in the 0.9% aqueous solution of NaCl at a potential scanning rate of 0.1 V/s: 1—the first cycle, 2—the fourth cycle.

**Figure 10:**
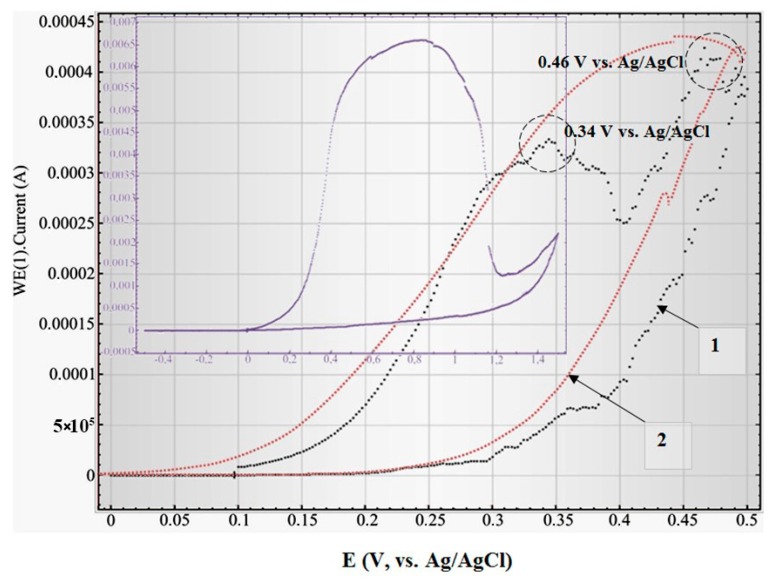
Influence of a scan rate of potential on electrochemical behavior (and cyclic polarization curve) of the Ti/PAAT/Ni electrode: 1—at a potential scanning rate 0.01 V/s (the first cycle). 2—at a potential scanning rate of 0.03 V/s (the first cycle). In the insert—at a scan rate of potential 0.1 V/s (the first cycle).

The results of the investigation of Ti/PAAT/Ni electrode electrochemical properties are represented in Figure 6, Figure 7, Figure 8, Figure 9 and Figure 10. Usually, the corrosion behavior of different composites and alloys is studied by the analysis of current-potential curves (voltammograms). The extrapolation of the line part of the cathode and anode polarization curve in semi-logarithmic (Tafel) coordinates to the E_corr_ [54,55] is considered to be the ordinary quick test to estimate the current density of corrosion.

This method is used when the electrochemical behavior of electrode materials is not accompanied by passivation, concentration polarization, and other abnormal effects. In the present case, the points of intersection of the anode and cathode polarization curves in Tafel coordinates (lg I–E) define the current of corrosion and the potential of corrosion (red lines in Figure 6).

Figure 6 shows the linear sweep voltammograms of the Ti/PAAT/Ni sample in semi-logarithmic coordinates. As the electrochemical deposition is performed into the porous matrix not up to a surface (on a certain depth), the top area of NiNPs (S_1_) is less than the visible surface area of the sample (0.36 cm^2^). In the inset is the scheme of such sample fragment cross-section. At a NiNPs density 10^10^ cm^−2^ and 70 nm pillar diameter S_1_ is equal to almost 0.15 cm^2^. Therefore, the current density J_corr_ has been determined for this area. S_2_ is the entire surface area, including the oxide surface area. This area is larger than S_1_. However, since we have previously shown that the oxide contributes insignificantly to the corrosion current, only the area of the S_1_ (area of the NiNPs tops) was considered in calculating the corrosion current density. Therefore, in Table 3 we showed the values of corrosion current density and samples corrosion current with the same “visible” surface area (electrolyte immersion area). In addition, in order to verify that the calculations were correct, we determined the corrosion current density in three ways: the polarization curves extrapolation method, the polarization resistance and Beleevskii methods). All methods produced comparable results.

As a rule, the free potential of corrosion stabilizes before the polarization processes. However, some experiments have been performed immediately after the immersion in the electrolyte and, in contrast, after several consecutive cycles of immersion of the just prepared samples. This enabled to obviate the passivation of Ni.

These experiments and Figure 6 showed that the conditions of performing the potentiodynamic tests affect the result. It can mean that the surface properties of a sample possibly change at anodic polarization. Therefore, a series of potentiodynamic experiments of identical Ti/PAAT/Ni electrodes was carried out right after the immersion and after the corrosion potential stabilizing.

For greater credibility, other known methods of corrosion analysis were employed: the polarization resistance method [54,55,56] and Beleevsky’s method [57].

Figure 7 presents a small part of the voltammogram (curve 1) in a limited range of potential (−10 to +10 mV) in order to determine polarization resistance *R_pol_*.

From this polarization curve slope and equation of Stern-Geary [58] the corrosion current can be determined:

(2) $$i_{corr}\;=\frac{1}{R_{pol}}\lfloor \frac{b_{a}\times \;b_{c}}{2.3\times (b_{a}+b_{c})}\rfloor$$

where *R_pol_* = 1ΔEΔI is polarization resistance at E = E*_corr_*.

The values *b_a_* and *b_c_* (Tafel slopes) have been estimated using the line portions of curve 2 near the potential of corrosion (pattern is shown in Figure 6 by red dashed lines) [59,60]. This mode is more accurate because it takes into account the probability of changing the properties of the sample surface during the measurement process.

Table 3 summarizes the corrosion parameters of two identical experimental samples No1 (curve 1, Figure 6) and No2 (curve 2, Figure 7), obtained in two ways: the polarization curves extrapolation (mode 1), and the polarization resistance mode (mode 2). The samples are identical, as were created in a batch fabrication, but differ only in conditions of polarization curves registration (the time of stay in the electrolyte): samples No1—fourth cycle of polarization (8 min), and samples No2—after the free potential of corrosion stabilizing before polarization test (20 min). Additionally, in Table 3 the following data is given: for bulk (metallurgical) Ni, flat electrodeposited Ni thick film, and nanocrystalline Ni thin film deposited by magnetron sputtering [61,62,63].

Via Beleevskii [57] method of three currents, the value of J*_corr_* = 0.34 µA/cm^2^ was received. The currents I_1_, I_2_, and I_3_ corresponding to π, 2π, and 3π polarization, which is rising in accordance with arithmetical progression. Magnitude π = E − E*_corr_*—is the sample polarization reference level relative to the free potential of corrosion.

It is visible from Table 3 that NiNPs surrounded by alumina are more chemically stable than bulk metallurgical Ni [63] and electrodeposited bulk Ni coating [61]. The corrosion currents for the studied samples are considerably lower. In addition, it is obvious that the corrosion resistance is higher than the bulk nanocrystal Ni produced by the electrochemical deposition [62], taking into account the sizes of crystallites and slightly different experimental conditions.

Further, the protective NiO layer growth on the top of the Ni pillars was demonstrated. In Figure 8, Figure 9 and Figure 10 cyclic voltammograms of the Ti/PAAT/Ni sample are shown. Potentiodynamic observation was performed at the range of potential of −1.4 to +1.4 V(Ag/AgCl) at a scanning rate of 0.10 V/s (Figure 8) and in a forward direction at a scanning rate of 0.01–0.03 V/s at the range of potential of 0 to 1.4 V(Ag/AgCl) (Figure 9 and Figure 10). The last mode of recording provides the stationary (near potentiostatic) growth of a passive layer on NiNPs top (to ensure the stability of the passive state) [64].

Besides, the experiment with the sample annealed at a temperature of 500 °C within 30 min has been performed. That is, the thin layer of Ni oxide on this sample was created in advance. The results for this sample are given in Supplemental File, Figure S1.

The electrochemical features of Ti/PAAT/Ni composite porous material differ from the reaction of an electrode with an ideal smooth surface and double electrical layer at the solution–sample interface for two reasons. Firstly, the ohmic resistance and reactions of Faraday affect the behavior of a composite material as a result of diffusion of the electrolyte inside the porous material with the developed surface (as evident from insets in Figure 8). In this Figure, a surface profile of NiNPs after partial selective etching of an oxide (Figure 8A) and the full surface profile of an experimental sample (Figure 8B) during electrochemical researches are shown. For example, one of the redox reactions of Faraday can happen on NiNPs surface because of transition Ni^2+^ to Ni^3+^ transition to form a thin layer of NiO on the NiNPs surface [64,65]. Secondly, electrode wettability also affects the result. Usually, a smooth metal surface with hydrophobic properties is poorly wetted with a weak water solution of NaCl. The uneven relief of PAAT with the long pores of nanosized diameter also has distinct wettability according to the preparation and experiment conditions [66]. Therefore, the wettability of the PAAT/NiNPs electrode surface must be controlled so as to attain more reproducible results. The oxide also has a different degree of wetting depending on the formation conditions.

On the anode branch at low potentials (Figure 8 and area I, red line on Figure 9) the Ni oxidation results in the formation of an oxy-hydroxide film, is recorded. Oxidation of Ni in the presence of Cl ions occurs according to the following reactions proposed in [67,68]:

Ni + H_2_O → Ni(H_2_O)_ads_ → Ni(OH)_ads_ + H_aqs_^+^ + e^−^ (3)

Ni(H_2_O)_ads_ + Cl^−^ → Ni(ClOH)^−^_ads_ + H^+^ + e^−^ (4)

Ni(OH)_ads_ + H^+^ → Ni^2^ + _aqs_ + (H_2_O)_ads_ + e^−^ → Ni(OH)_2_ (5)

The main reaction, occurring at the anode at high potentials (sections III, IV, blue line on Figure 9) is the oxy-hydroxide film breakdown [64]. It is mainly the result of chlorine ion penetration through the thin oxyhydroxide film. On the cathode branch of the polarization curve (Figure 8) the irreversible wave of electrochemical reduction of Ni^+2^ ions has occurred at potential values E = −(1100–1250) mV vs. Ag/AgCl. The following reactions are possible on the electrode:

NiO + 2H^+^ + 2e^−^ → Ni + H_2_O (6)

And/or Ni(OH)_2_ + 2H^+^ + 2e^−^ → Ni + 2H_2_O (7)

On an anode curve (Figure 9) the specific (characteristic) regions of active dissolution (I), transition of active-passive (II), passivity (III) and transpassivity (IV) are observed. In the region of active dissolution, Ni passes into the solution in the form of bivalent ions of Ni^2+^. In the region of active-passive transition, the decrease of current is caused by the NiO thin film growth on the surface of the metal. The current plateau is clearly associated with metallic oxidation [30]. In the transpassive area, as well as in the active dissolution region, Ni passes into the solution in the form of Ni^2+^ [65,69]. The current of transpassive dissolution is less than the current of active dissolution. It barely changes at return cycling. It means that the passive film of Ni does not exfoliate and becomes denser on-cycling which assists in improving this material’s corrosion resistance.

The pre-treated samples (after stabilizing of corrosion potential, Figure 7 or after annealing, Figure S1) have exhibited active-passive polarization behavior. As revealed in [70], a low scanning rate and suitable scanning range allow increasing electron transfer reactions in the sample and thus leading to a cyclic polarization curve. At a low rate of anode polarization (0.01 V/s), the anode current in the range from 0 to 0.5 V (Ag/AgCl) (area I in Figure 9) changes as it is testified in Figure 10.

At this detection rate, several peaks of oxidation were fixed: at 0.34 V (Ag/AgCl) and 0.46 V (Ag/AgCl). Hypothetically, the current grows until the continuous solid layer of highest valency oxide (Ni_2_O_3_) is formed on the external border of the lowest valency oxide film (NiO) [71]. After the formation of Ni_2_O_3_ (at 0.47 V (Ag/AgCl)), the anode current is reduced practically up to zero at 0.2 V (Ag/AgCl), which is caused by screening the lowest valency oxide from the solution with a layer of the highest valency oxide. The latter has a stoichiometric composition and is an insulator. Thus, the two peaks of oxidation can be connected with the consecutive formation of various Ni oxide layers on the Ti/PAAT/Ni electrode surface. That is, it’s likely that there is two-level oxidation of Ni: at the beginning to the oxidation level +1, and then up to +2.

The similar mechanism of Ni oxides formation on a usual, continuous film of electrochemical Ni was studied in the electrolyte on the basis of NaOH, and redox peaks were attributed to transitions firstly from Ni to NiO and then to NiOOH [72,73].

In work [62] another mechanism of a protective film formation on the electrochemical nanocrystalline Ni surface is offered. In this work the corrosion research was performed in three aqueous solutions: 10% NaOH, 3% NaCl and 1% H_2_SO_4_. The growth of Ni (OH)_2_ passive layer in anode polarization can include the following electrochemical and chemical stages [62]:

Ni + H_2_O → Ni(H_2_O)_ads_ (8)

Ni(H_2_O)_ads_ → Ni(OH)^+^ + H^+^ + 2e^−^ (9)

Ni(OH)^+^ + OH^−^ → Ni(OH)_2_ (10)

Ni(OH)_2_ → NiO + H_2_O (11)

At first, Ni hydroxide (the Equations (8)–(10)) is formed in all electrolytes. Then NiO is formed (the Equation (10)) only in the NaCl aqueous solution [62].

In other research works, similar options are offered, but there is no uniform point of view on this matter yet. The established feature of Ni corrosion processes is the participation of free electrons. Therefore, the rate of such processes is proportional to the number of electrons that are taken away from the metal surface in a unit of time. Consequently, the corrosion rate is a linear function of corrosion current.

Thus, there is no doubt that the passive film (oxy-hydroxide layer) is formed on the NiNPs tops in the course of research. There is just no uniform point of view on the mechanism of this film formation. To a large extent, it can be connected with slightly different experiment conditions: environment parameters, rate and range of scanning, surface condition of a sample (its prehistory), etc. The authors of work [74,75] consider that surface defects of the crystal lattice of electrochemical Ni are the centers of passive film nucleation.

In our case the corrosion resistance of NiNPs in PAAT is provided (1) due to the PAAT protective properties and (2) because of the fast growth of Ni oxide layer on the tops of NiNPs, inhibiting the penetration of active ions of hostile environment and dissolution.

## 4. Conclusions

Disclosed is a mode of forming composite material NiNPs in porous alumina-based on low-temperature electrochemical operations. The method of composite material creation using low-temperature reactions is presented. As revealed by the XRD analysis the Ni-alumina nanocomposite material had poly-nanocrystalline nature and the main grain size about 18 nm.

The results of cyclic and linear sweep voltammograms studying were offered via two ways: Tafel extrapolation of the cathode and anode polarization curves and by determining polarization resistance. Both methods yield comparable results.

The pre-treated samples (after stabilizing of corrosion potential or after annealing) have shown active-passive polarization behavior. The corrosion potential and zero current potential shift towards the positive direction and lower corrosion current densities have been related to the modification in the anodic processes (redox reactions as a result of Ni^2+^ to Ni^3+^ transition to form a thin film of NiO on the NiNPs surfaces). From the Tafel plots, the currents of corrosion are much lower and corrosion resistance is higher in comparison with metallurgical Ni or electrodeposited thick Ni films. Investigations at a small scanning rate and corresponding scanning range allowed recording the fact of NiO film formation on the surface of NiNPs.

Surface topological microstructure and surface wettability of the sample (in our case composite material) subjected to corrosion testing also have an influence on the result. The received data reveals that PAAT is both a forming template and reliable corrosion protection. Thus, SEM and electrochemical investigations testify the corrosion resistance of Ti/PAAT/NiNPs composite also due to the protective properties of PAAT in a large potential range.

The results of the corrosion study of Ti/PAAT/NiNPs nanocomposite material will allow us to optimize its formation technology. It will be also useful to forecast the long-time stability of such devices as biosensors sensitive elements, magnetic memory elements, nano-emitters in liquid crystal display devices, etc.

In order to determine the possibilities of using these nanostructures in the field of targeted drug delivery, it is necessary to separate NiNPs (magnetic carrier) from the substrate and carry out studies of magnetic properties and cytotoxicity, as described in one of the previously published works [17]. Spherical nanoparticles are commonly used for this purpose. The novelty of these studies is that instead of spherical particles we are going to use NiNPs with similar size and properties. This is achieved by the template synthesis method. However, not only the size of the nanoparticles but also the aspect ratio is important for application in this particular field. At the moment we are in the process of determining the optimal size of structures, and the results of the research will be published in the near future.

In summary, the research has shown that the obtained Ni-alumina nanocomposite has high corrosion resistance in −450 to +450 mV (Ag/AgCl) range of potentials in the examined solution, which has found wide application in corrosion and biomedical investigations of advanced nanostructured materials.

## Figures and Tables

**Table 1:** Topological characteristics of NiNPs.

Characteristic	Value
Diameter of pillars d	(50–75) ± 10 [nm]
Length between pillars D	105 ± 10 [nm]
Pillars height h, [nm]	(400–1000) ± 15
Aspect ratio (h/d)	5–20
Pillars density N, [per cm^2^]	5 × 10^9^–1.5 × 10^10^

**Table 2:** The main properties of the XRD pattern of Ni in PAAT.

Crystal Orientation	2 Theta, [deg.] *	Intensity I, [%]	Size of Crystallites D, [nm]
Ni (111)	43.82(44.51)	100	18
Ni (200)	51.07(51.85)	8.9	9
Ni (220)	74.90(76.37)	3.2	20

* 2 Theta in brackets are the values from the JCPDS database.

**Table 3:** The Ti/PAAT/Ni nanocomposite corrosion parameters, determined by two ways: the polarization curves extrapolation (mode 1) and using the polarization resistance (mode 2).

Sample No and Mode of Test	*E_corr_*, V (Ag/AgCl)	*I_corr_*, [µA]	*J_corr_*, [µA /cm^2^]	*b_a_*, [mV/decade]	*b_c_*, [mV/decade]	*R_p_*, [Ω/cm^2^]
No1 Ti/PAAT/Ni (1st mode)	−0.213	0.065	0.433	‒	‒	‒
No1 Ti/PAAT/Ni (2nd mode)	−0.213	0.091	0.610	193	155	1.25 × 10^6^
No2 Ti/PAAT/Ni (1st mode)	−0.265	1.22	0.339	‒	‒	‒
No2 Ti/PAAT/Ni (2nd mode)	−0.265	1.65	0.458	172	162	0.61 × 10^6^
Ni [61] ^1^ bulk electrodeposited	−0.303	‒	2.226	185	273	‒
Ni [62] ^2^ bulk nano electrodeposited	−0.494	‒	5.210	‒	‒	‒
Ni [63] ^3^ bulk metallurgical	−0.428	–	7.059	‒	‒	‒

^1^ electrodeposited bulk Ni (3% NaCl), ^2^ electrodeposited nanocrystalline (size of grain 16 nm) Ni (3% NaCl), ^3^ bulk metallurgical Ni (99.85%) with size of grain 47.32 µm (1 M NaCl).

## Supplementary Materials

The following are available online at https://www.mdpi.com/2079-4991/10/1/173/s1, Figure S1: Cyclic voltammogram of the Ti/Al_2_O_3_/Ni sample in the 0.9% aqueous solution of NaCl at a potential scanning rate of 0.1 V/s after annealing at 500 °C for 30 min (the third full cycle).
