# Supplementary material for: Electrochemical Behaviour of Ti/Al2O3/Ni Nanocomposite Material in Artificial Physiological Solution: Prospects for Biomedical Application
Source: Nanomaterials (Basel). 2020 Jan 19;10(1):173. doi: 10.3390/nano10010173 (PMC7022230; doi:10.3390/nano10010173)
Supplement: Supplementary file 1 [file nanomaterials-10-00173-s001.pdf]

Supplemental File

Electrochemical Behaviour of Ti/Al_2O_3/Ni Nanocomposite Material in Artificial Physiological Solution: Prospects for Biomedical Application

Alla Vorobjova [1], Daria Tishkevich [2,3,*], Dmitriy Shimanovich [1], Maxim Zdorovets [4,5,6], Artem Kozlovskiy [4], Tatiana Zubar [2,3], Denis Vinnik [3], Mengge Dong [7], Sergey Trukhanov [2,3], Alex Trukhanov [2,3] and Valery Fedosyuk [2]

1 Department of Micro- and Nanoelectronics, Belarusian State University of Informatics and Radioelectronics, 220013 Minsk, Belarus; vorobjova@bsuir.by (A.V.); shdl@tut.by (D.S.)

2 Laboratory of Magnetic Films Physics, Scientific-Practical Materials Research Centre of National Academy of Sciences of Belarus, 220072 Minsk, Belarus; fix.tatyana@gmail.com (T.Z.); sv_truhanov@mail.ru (S.T.); truhanov86@mail.ru (A.T.); fedosyuk@physics.by (V.F.)

3 Laboratory of Single Crystal Growth, South Ural State University, 454080 Chelyabinsk, Russia; denisvinnik@gmail.com

4 The Institute of Nuclear Physics, Almaty 050032, Kazakhstan; artem88sddt@mail.ru (A.K.); mzdorovets@gmail.com (M.Z.)

5 L.N. Gumilyov Eurasian National University, Nur-Sultan 010008, Kazakhstan

6 Ural Federal University named after the First President of Russia B.N. Yeltsin, 620075 Yekaterinburg, Russia

7 Department of Resource and Environment, Northeastern University, Shenyang 110819, China; mg_dong@163.com

* Correspondence: dashachushkova@gmail.com; Tel: +375-2956-28187

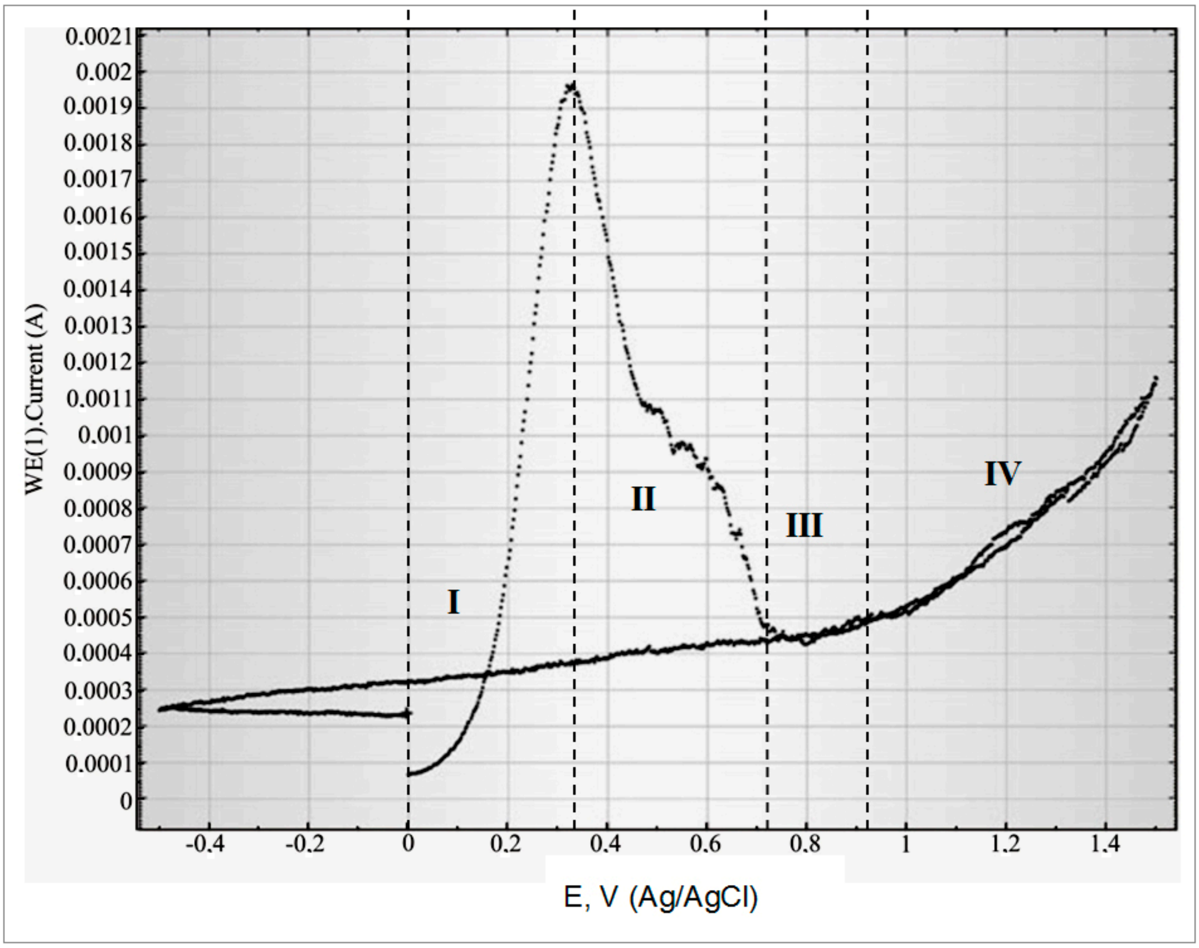

Figure S1. Cyclic voltammogram of the Ti/Al_2O_3/Ni sample in the 0.9% aqueous solution of NaCl at a potential scanning rate of 0.1 V/s after annealing at 500 °C for 30 min (the third full cycle).
